# A Novel Network Integrating a miRNA-203/SNAI1 Feedback Loop which Regulates Epithelial to Mesenchymal Transition

**DOI:** 10.1371/journal.pone.0035440

**Published:** 2012-04-13

**Authors:** Michèle Moes, Antony Le Béchec, Isaac Crespo, Christina Laurini, Aliaksandr Halavatyi, Guillaume Vetter, Antonio del Sol, Evelyne Friederich

**Affiliations:** 1 Cytoskeleton and Cell Plasticity Lab, Life Sciences Research Unit-FSCT, University of Luxembourg, Luxembourg, Luxembourg; 2 Luxembourg Centre for Systems Biomedicine, University of Luxembourg, Esch-sur-Alzette, Luxembourg; University of Colorado, Boulder, United States of America

## Abstract

**Background:**

The majority of human cancer deaths are caused by metastasis. The metastatic dissemination is initiated by the breakdown of epithelial cell homeostasis. During this phenomenon, referred to as epithelial to mesenchymal transition (EMT), cells change their genetic and trancriptomic program leading to phenotypic and functional alterations. The challenge of understanding this dynamic process resides in unraveling regulatory networks involving master transcription factors (e.g. SNAI1/2, ZEB1/2 and TWIST1) and microRNAs. Here we investigated microRNAs regulated by SNAI1 and their potential role in the regulatory networks underlying epithelial plasticity.

**Results:**

By a large-scale analysis on epithelial plasticity, we highlighted miR-203 and its molecular link with SNAI1 and the miR-200 family, key regulators of epithelial homeostasis. During SNAI1-induced EMT in MCF7 breast cancer cells, miR-203 and miR-200 family members were repressed in a timely correlated manner. Importantly, miR-203 repressed endogenous SNAI1, forming a double negative miR203/SNAI1 feedback loop. We integrated this novel miR203/SNAI1 with the known miR200/ZEB feedback loops to construct an *a priori* EMT core network. Dynamic simulations revealed stable epithelial and mesenchymal states, and underscored the crucial role of the miR203/SNAI1 feedback loop in state transitions underlying epithelial plasticity.

**Conclusion:**

By combining computational biology and experimental approaches, we propose a novel EMT core network integrating two fundamental negative feedback loops, miR203/SNAI1 and miR200/ZEB. Altogether our analysis implies that this novel EMT core network could function as a switch controlling epithelial cell plasticity during differentiation and cancer progression.

## Introduction

Carcinomas arise in epithelial tissues and the metastatic cascade is initiated by the breakdown of epithelial cell homeostasis. During this transient phenomenon, referred to as epithelial to mesenchymal transition (EMT) which also occurs during embryonic development, cells lose their epithelial features, including cell-cell adhesions and cell polarity, and gain cell motility, mesenchymal and stem cell-like properties. EMT can be initiated by multiple pathways converging in the activation of EMT inducers, such as SNAI1/2, ZEB1/2 and TWIST1, transcription factors which repress epithelial-specific genes [1], [2].

MicroRNAs (miRNAs) are short noncoding RNAs that post-transcriptionally control gene expression through imperfect base-pairing to the 3′ untranslated region (3′UTR) of target messenger RNAs. MiRNAs recently emerged as important regulators in EMT, the most prominent being the two clusters of the miR-200 epithelial marker family: miR-200b/200a/429 (miR-200b) and miR-200c/141 (miR-200c) [3], [4]. The miR-200s regulate EMT through a double negative feedback loop with the ZEB factors, which, depending on the relative levels of miR-200 and ZEB, can direct the switch from epithelial- to mesenchymal-like states and back [5]–[8]. In addition, the transcription factor SNAI1, which plays a key role during the early steps of EMT, activates the expression of ZEB factors in a context-dependent manner [1], [9]–[12]. An integrated view, on how these transcription factors and miRNAs contribute together to regulatory networks acting as switches between epithelial and mesenchymal states, is however lacking. The dynamic properties of such networks [13], [14] are affected notably through feedback loops involving miRNAs and transcription factors acting as toggle switches [15], [16].

Here, we performed a large-scale analysis highlighting miR-203 as consistently associated with epithelial plasticity and correlated to the miR-200 family which plays a key role in epithelial homeostasis. Furthermore, our experimental data connected miR-203 and the transcription factor SNAI1 in a double negative feedback loop. Based on our present and published data, we integrated this novel miR203/SNAI1 and the well-characterized miR200/ZEB feedback loops into a SNAI1-orchestrated EMT core network. Dynamic simulation revealed the existence of two stable states for this network and showed that the miR203/SNAI1 loop plays a crucial role in the switch from an epithelial to a mesenchymal state and in the stabilization of the core network in these two states. These findings support previous studies [13], [17] showing the key role of feedback loops in network stability and determination of cell fate and plasticity.

## Results and Discussion

### MiR-203 is associated with SNAI1 and the miR-200s

**Figure 1:**
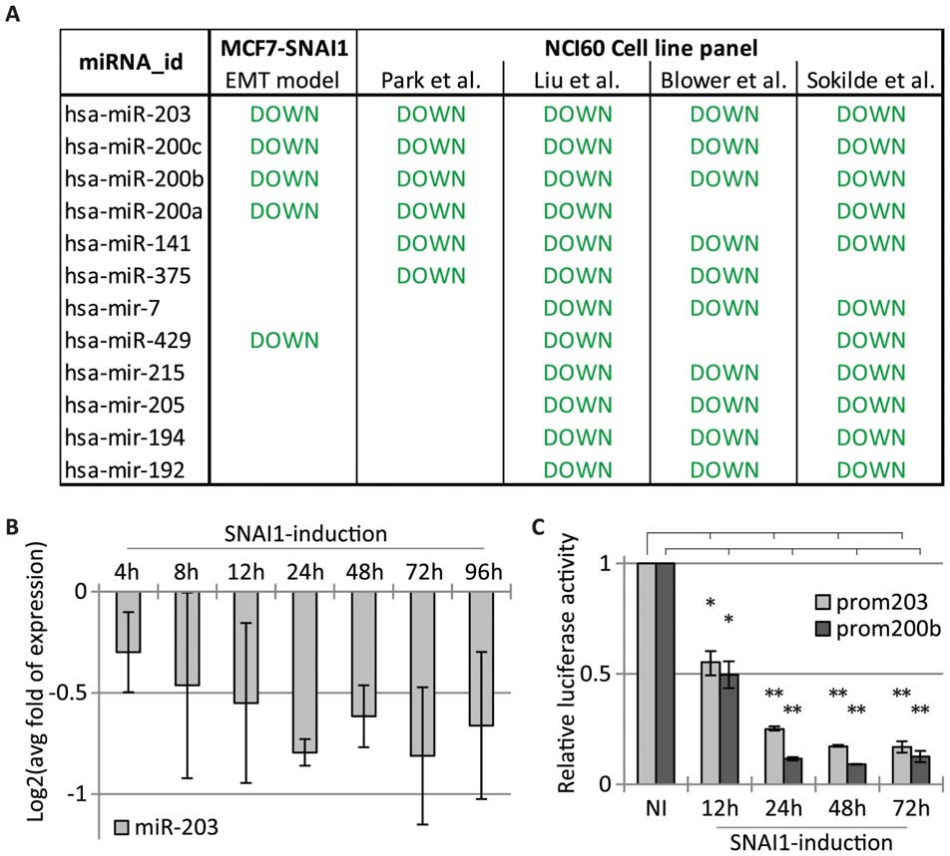
Large-scale analysis of miRNA expression signatures, and miR-203 expression during SNAI1 induction in MCF7-SNAI1 cells. A) List of miRNAs found downregulated, in at least three studies, in the large-scale analysis. B) qRT-PCR analyses of miR-203 expression levels normalized to U44 expression and expression levels in non-induced cells. C) Relative luciferase activity of miR-203 and miR-200b promoter constructs in non-induced (NI) and SNAI1-induced cells. Data are normalized to “NI” (*, p<0.05; **, p<0.01).

To identify miRNAs participating in SNAI1-orchestrated regulatory networks, we analysed our time-resolved microarray data (GEO accession: GSE35074) of EMT, triggered by conditional expression of SNAI1 in “Tet-Off” MCF7-SNAI1 breast carcinoma cells [18], [19]. At an established EMT state, 61 miRNAs were differentially expressed (Table S1). Among those, 29 miRNAs were repressed and potentially regulated by the transcriptional repressor SNAI1. We combined these experimental results with miRNA expression signature analyses of four published datasets of epithelial and mesenchymal NCI60 cancer cell lines (Fig. 1A, Table S2) [20]–[23], and calculated expression correlations with the miR-200 epithelial marker family (Table S3). Interestingly, these analyses highlighted miR-203, whose expression was downregulated in our EMT model and mesenchymal cancer cell lines, as well as highly correlated to the expression of the miR-200s. Co-regulated overexpression of miR-203 and miR-200 family members has been reported during early stages of human stem cell differentiation into epidermal cells suggesting their participation to this process [24]. Conversely, miRNA expression analysis in endometrial carcinosarcomas, a *bona fide* example of EMT *in vivo*, revealed a marked downregulation of miR-203 and miR-200 family members in the mesenchymal areas, concomitant to the upregulation of EMT inducers, including SNAI1 [25]. Further expression profiling in human primary and metastatic cancers showed that miR-203 and miR-200 family members were significantly suppressed in the latter, suggesting a direct involvement in cancer metastasis [26]. These large-scale analyses indicated that these molecular actors may work together, as positive or negative regulators. Therefore, we decided exploring the regulation of miR-203 and miR-200 family members through SNAI1, and their integration into regulatory networks governing epithelial cell plasticity.

### SNAI1 represses miR-203 and miR-200b expression

First, we assayed miR-203 expression during SNAI1 induction in our EMT model by qRT-PCR analyses. MiR-203 was continuously repressed upon SNAI1 induction, similarly to the miR-200b cluster (Fig. 1B; Fig. S1). We further showed that miR-203 and miR-200b promoter activity significantly decreased upon 12 h of SNAI1 induction (Fig. 1C). In line with our findings, overexpressed SNAI1 reduced promoter activity of miR-203 [27], and of the miR-200c cluster [7], [27], in a HEK293T and HCT116 cell system, respectively. Also, SNAI1 has been shown to repress miR-200 family members during murine embryonic stem cell differentiation [28]. In addition, in line with previous results [9], [10], overexpression of SNAI1 in our MCF7-SNAI1 cell model induced an upregulation of ZEB1 (our unpublished observation) which targets miR-203 and the miR-200s [6], [7], [27]. Altogether these data suggest that SNAI1 regulates expression of miR-203 and both miR-200 clusters in a coordinated manner, and that these miRNAs co-act in SNAI1-regulated programs.

### MiR-203 downregulates endogenous SNAI1 and promotes epithelial-like properties in breast cancer cells

**Figure 2:**
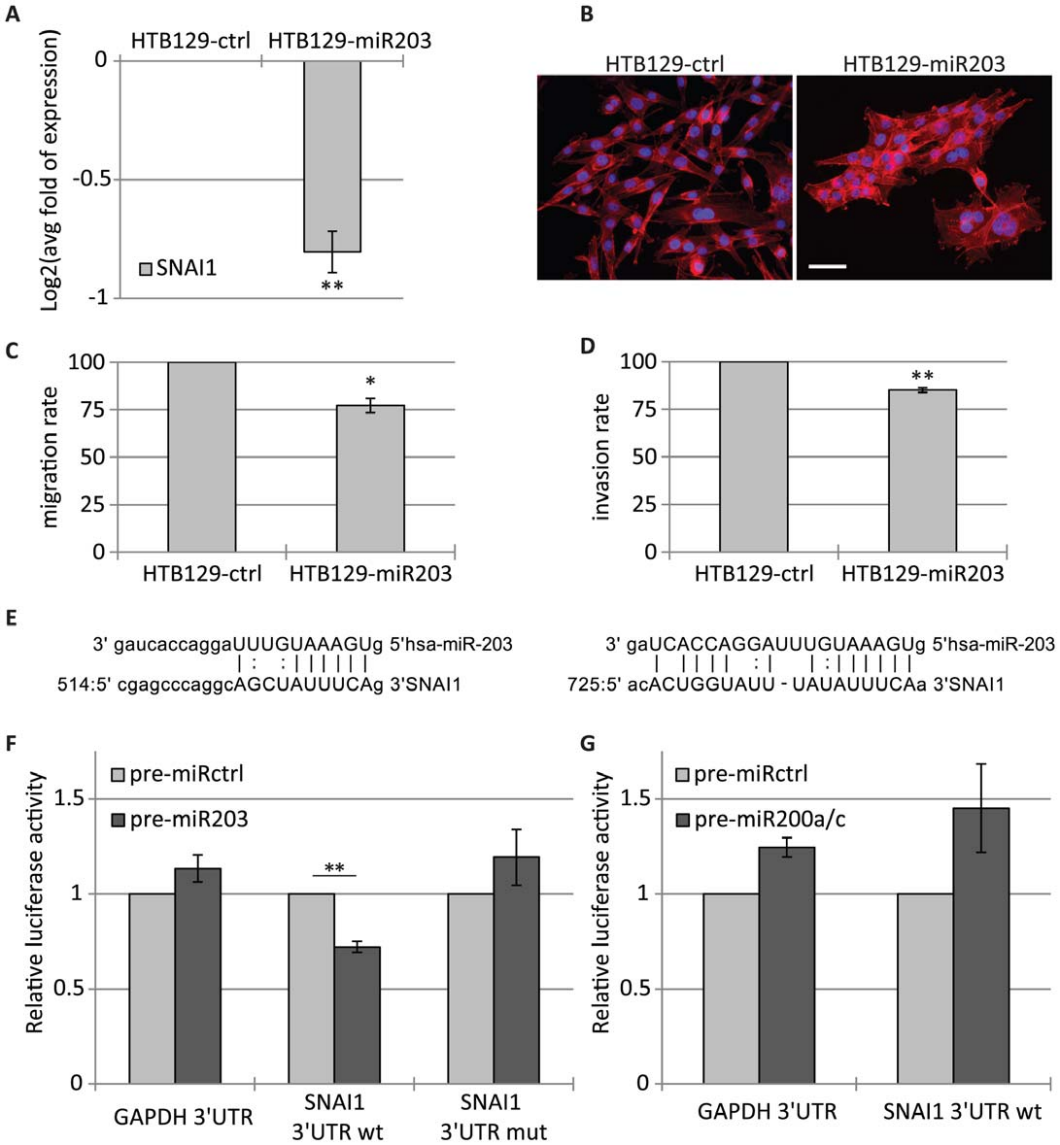
MiR-203 promotes epithelial-like features and represses endogenous SNAI1. HTB129-ctrl and HTB129-miR203 cells were subjected to A) qRT-PCR analyses of SNAI1 mRNA expression in HTB129-ctrl and HTB129-miR203 cells, B) fluorescent staining with DAPI (blue) and phalloidin (red) (scale bar, 20 µm), C) migration and D) invasion assays. E) Predicted miR-203 target sites within SNAI1 3′UTR. F, G) Relative luciferase activity of SNAI1-3′UTR wild type (wt) or mutant (mut) in MDA231 cells transfected with control (F, G), miR-203 (F) or miR-200a/c (G) precursors. Co-transfection with GAPDH 3′UTR vector served as additional negative control (*, p<0.05; **, p<0.01).

Next, we investigated the role of miR-203 in relationship with SNAI1 expression in breast carcinoma cells. In MCF7-SNAI1 cells, ectopically expressed SNAI1 lacks its natural 3′UTR [18] and therefore, these cells are not suitable to study whether miR-203 regulates SNAI1 messenger RNA (mRNA). The mesenchymal breast cancer cell line HTB129 presents high levels of endogenous SNAI1 and low levels of miR-203 as compared to epithelial MCF7 cells [21], [29]. HTB129 cells stably transfected with miR-203 (HTB129-miR203) exhibited a significant decrease in SNAI1 mRNA (Fig. 2A, Fig. S2). HTB129-miR203 cells lost their typical fibroblastic, dispersed phenotype and acquired a more compact and cohesive appearance (Fig. 2B). HTB129-miR203 cells further lost about 25% of their migratory and 15% of their invasive capacity (Fig. 2C, D). By performing MTT proliferation and AnnexinV apoptosis assays we excluded that observed inhibitions were due to decreased cell proliferation and/or programmed cell death (Fig. S3). Interestingly, previous studies in prostate cancer progression and metastasis showed that miR-203 expression not only controlled cell migration and invasion of prostate cancer cell lines, but also suppressed prostate cancer metastasis *in vivo* via repression of prometastatic targets such as ZEB2 [30], [31]. HTB129 cells expressed high levels of ZEB1/2 factors, but in the present cellular context, miR-203 expression did not lead to significant decrease of ZEB2 mRNA. This may be due, in part, to incomplete miR-203-mediated repression of SNAI1 which has been shown previously to promote upregulation of ZEB factors [32]. In addition, HTB129 cells co-expressed high levels of SNAI2 and TWIST1 (our unpublished data), two EMT inducers. Collectively, these factors may attenuate the effects of miR-203-mediated repression of SNAI1 in HTB129-miR203 cells. Accordingly, we did not detect a significant increase in expression of classical epithelial markers such as E-cadherin and keratin 18 in these cells (our unpublished observations). Altogether these results show that miR-203 significantly reduces SNAI1 expression and promotes epithelial-like features such as a more cohesive phenotype and reduced motility, motivating us to investigate whether miR-203 could directly target SNAI1.

### MiR-203, but not the miR-200s, directly represses SNAI1

*In silico* analysis predicted two binding sites for miR-203, but none for miR-200 family members, within the 3′UTR of the SNAI1 mRNA (Fig. 2E) (microRNA.org, August 2010 Release) [33]. The ability of miR-203 to directly target SNAI1 was evaluated by luciferase reporter assays in MDA231 cells, using SNAI1-3′UTR reporter constructs - wild type or lacking the predicted miR-203 target sites. Overexpression of miR-203 in MDA231 cells reduced the activity of the wild type SNAI1-3′UTR, but not the mutant construct (Fig. 2F). Further, in agreement with *in silico* predictions, miR-200a and miR-200c (miR-200a/c), representing both seed sequences found within the miR-200 family, did not repress wild type SNAI1-3′UTR reporter activity (Fig. 2G). A similar result was obtained in the unrelated HeLa cell line (data not shown). These results indicate that miR-203, but not the miR-200s, directly regulates SNAI1 expression, thus linking miR-203 and SNAI1 in a double negative feedback loop and suggesting convergent yet not identical roles for these miRNAs in the regulation of SNAI1-orchestrated processes.

### Integration of miR203/SNAI1 in an EMT core network

**Figure 3:**
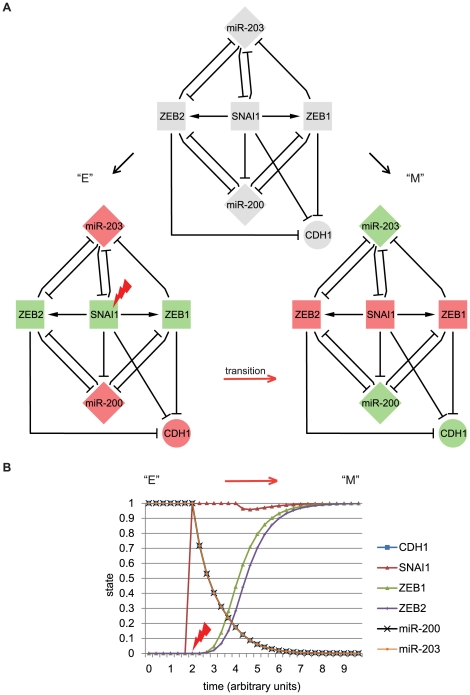
EMT core network integrating the miR203/SNAI1 and miR200/ZEB double negative feedback loops. A) The top panel corresponds to the core network integrating described interactions between miR-203, miR-200s (miR-200), SNAI1, ZEB1, ZEB2 and E-cadherin (CDH1). The bottom panels show the stable states “E” and “M” obtained after dynamic analyses. B) *In silico* upregulation of SNAI1 in a continuous dynamic system of the EMT core network. The state of SNAI1 is changed from “0” to “1” at time point 2 (arbitrary units of time), during two units of time. Diamonds represent miRNAs, squares transcription factors, and circles coding-genes other than transcription factors. Red and green colours stand for upregulated and downregulated expression levels, respectively. Edges represent an interaction between two actors, either activation (arrow) or inhibition (blunt arrow). The “lightning” indicates a SNAI1 upregulation triggering the transition from state “E” to “M” (red arrow).

We integrated the novel miR203/SNAI1 feedback loop together with the known miR200/ZEB feedback loops [5] into an *a priori* SNAI1-centered EMT core network (Fig. 3A), based on our present and published data. SNAI1 represses the transcription of both miR-200 clusters (our data) [7], [27], [28] and indirectly activates expression of the ZEB factors (our unpublished observation) [9]–[11]. Further, ZEB1/2 inhibit miR-203 promoter activity [27] and in turn miR-203 targets and represses ZEB2 [30]. E-cadherin which is directly repressed by the SNAI1 and ZEB factors [1], was added to the network as an epithelial target gene.

Dynamic simulation of our core network revealed two stable states which we associated with an epithelial “E” and mesenchymal “M” phenotype as described in literature (Fig. 3A, Data S1) [1]. Transition probability further attributed a high robustness to both states (Data S1), implicating that the core network is unlikely to switch between states without external stimulus. Importantly, the simulation of an upregulation of SNAI1 triggered the transition from state “E” to “M” (Fig. 3A, B). Next, to show the importance of the miR203/SNAI1 feedback loop on the network dynamics, we performed an ‘edgetic’ (edge-specific genetic) perturbation, by removing the “miR-203 on SNAI1” interaction [34]. Interestingly, the dynamic simulation of the edge-altered core network revealed a single stable state “E_ea_” (edge-altered state “E”) (Data S1). Thus, the feedback regulation “miR-203 on SNAI1” is crucial for switching from an epithelial to a mesenchymal state and in stabilising the core network in both states.

### Conclusion

Co-expression of EMT master regulators in cancer cells makes it difficult to understand the molecular hierarchy and cooperation between them. Our dynamic MCF7-SNAI1 cell model allowed evaluating the link between SNAI1 expression and other molecular actors involved in EMT, which we further analysed in HTB129 and MDA231 cells. Collectively, our integrative study implies that this core network (Fig. 3) could function as a robust switch controlling early steps of EMT and epithelial homeostasis, further emphasizing the importance of bistable feedback loops in determining cell plasticity [13], [17]. Obviously, the present core network is embedded into a larger network with multiple molecular actors such as SNAI2 and TWIST1 (our observations) the expression of which is interconnected in a context-dependent manner and regulated by various pathways [1], [32], [35]. This core network will provide a good starting point to further study key regulatory circuits underlying EMT, such as TGFβ signalling which plays an important role in transient cancer cell invasion. Indeed, TGFβ induces EMT via upregulation of SNAI1 at early states of the transition while subsequent expression of ZEB1/2, SNAI2 and TWIST1 may maintain the mesenchymal, migratory phenotype [35], [36]. Interestingly, in addition to ZEB1 also SNAI2 and TWIST1 increased during SNAI1-induction in MCF7-SNAI1 cells (our unpublished observations). Based on previous findings, it is likely that in our cell model, the transcriptional repressors SNAI1 and SNAI2 may work in concert and target common genes via binding to E-boxes of their promoters [1]. In future, further dissection of the molecular links between EMT master regulators and miR-200s/miR-203, together with the consideration of quantitative binding parameters, will allow completing our core network and will contribute to the better understanding of key regulatory circuits underlying EMT.

## Materials and Methods

### Epithelial/mesenchymal miRNA expression signature study

Data from the NCI60 panel were analyzed using the same statistical analysis (t-test) and classification as described in Park et al [21]. Differentially expressed miRNAs were filtered using a p-value threshold of 0.01. Expression levels (UP or DOWN) correspond to the sign of the difference between the average of log-intensity values of mesenchymal cells and the average of log-intensity values of epithelial cells: 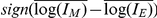.

### MiRNA microarray analysis

MiRNA microarray design, protocols and data have been deposited in NCBI's Gene Expression Omnibus and are accessible through GEO Series accession number GSE35074. The established state of EMT is considered reached after 72 h to 96 h of SNAI1 induction and refers to the “late EMT stage” we previously defined by analyzing transcriptional events as well as phenotypic changes occurring upon SNAI1 induction in our MCF7-SNAI1 EMT model [18]. Averaged expression values for each time point (72 h and 96 h) were calculated taking into account only replicates which have moduli of log-ratios≥0.5 and t-test p-values≤0.01 (according to LCSciences data processing).

### Vector constructs

For exogenous miR-203 expression, hsa-miR-203 stem-loop sequence (MI0000283) −200/+192 relative to the first and last nucleotide of the stem-loop, was synthesized and cloned into *Bgl*II/*Hin*dIII sites of the pSUPER.retro.puro vector (pSUPER-miR-203) (OligoEngine) (DNA2.0). Hsa-miR-203 promoter region [27] was synthesized and cloned into pGL3-basic reporter using *Kpn*I/*Hin*dIII sites (DNA2.0). Wild type human GAPDH- and SNAI1-3′UTR, and mutant SNAI1-3′UTR lacking the predicted miR-203 binding sites, were synthesized and cloned into the psiCHECK™-2 (Promega) vector at *Xho*I/*Not*I sites (DNA2.0). Mir-200b promoter construct, pGL3miR200b/200a/429 (−321/+120) has been previously described [6].

### Cell lines

“Tet-Off” MCF7-SNAI1 cells expressing human SNAI1 upon removal of tetracycline from the culture medium have been previously described [18], [19]. The human breast cancer cell lines HTB129 and MDA231 (also named MDA-MB-231 or HTB-26), purchased from the ATCC, were maintained in RPMI1640 and Leibovitz culture media (Lonza), respectively, supplemented with 10% fetal bovine serum, 2 mM L-glutamine, 100 U/ml penicillin and 100 µg/ml streptomycin. HTB129 cells stably expressing miR-203 were generated by pSUPER-miR203 vector transfection and puromycin selection. Cells stably transfected with the empty pSUPER.retro.puro vector served as control.

### Epifluorescence staining of cells

To reveal and illustrate the cell phenotype, DNA and F-actin were stained with DAPI (MPBiochemicals) and Phallo504 (Invitrogen), respectively. Cells were analyzed by epifluorescence microscopy (Leica DMRX microscope). Images were acquired with a linear CCD camera (Micromax) and analyzed with Metaview software (Universal Imaging Corporation Ltd).

### RNA extraction and real-time quantitative PCR (qRT-PCR)

Total RNA was extracted using Trizol as recommended by the manufacturer (Invitrogen). RNA quality and concentration were evaluated spectroscopically using a NanoDrop 2000c instrument (ThermoScientific). Reverse transcription and qRT-PCR quantification of miRNA and mRNA were carried out as described previously [18], [37], U44 and GAPDH served as internal references, respectively. Oligonucleotides used in this study are listed in Table S4.

### Luciferase reporter assays

Indicated cell lines were plated in 6-well plates and transfection was carried out using Lipofectamine 2000 (Invitrogen). For promoter reporter assays, cells were cotransfected with a pGL3-promoter construct (600 ng) and a pRL-TK reference plasmid (5 ng) (Promega). For 3′UTR reporter assays, cotransfection was realized with 90 ng 3′UTR-psiCHECK™-2 constructs and a total of 75 pmol Pre-mir™ miRNA Precursor Molecules (Ambion). After 24 h of incubation cells were lysed, and firefly and *Renilla* luciferase activities were measured with a FluoStar Optima instrument (BMG LABTECH) using the Dual-Luciferase Reporter Assay System (Promega). All reporter assays are shown as relative luciferase activities, normalized to controls.

### Cell migration assay

Cell migration was evaluated using Ibidi culture inserts according to the manufacturer's protocol (Ibidi). Cells were seeded into the Culture-Inserts and grown overnight to confluency. After removal of the insert a 500 µm cell-free gap was created. Phase contrast images of the same gap fields were captured at 0 h and 24 h of incubation using an inverted light microscope (Leica DMIL) with camera (Leica DFC360 FX). Gap closure was quantified using ImageJ software (NIH).

### Cell invasion assay

5×10^4^ cells were seeded in 2% FBS medium, onto Transwell plates coated with 50 µg of extracellular matrix proteins (ECM gel E1270, Sigma). 10% FBS medium was added to the lower chamber as chemoattractant. After 24 h, cell invasion was quantified using the MTT assay (Sigma).

### Statistical analysis

Assays were performed in technical triplicates and repeated in at least three biological replicates. Presented data are mean ± SEM of three biological replicates. Paired t-test was used to estimate p values. For the 3′UTR reporter assays one-tailed paired t-test was used to check for a potential decrease in relative luciferase activity. p<0.05 was considered to be statistically significant. For qRT-PCR assays, Log2-transformed mean fold changes (averaged over three biological replicates) are presented. Error bars are the SEM recalculated using the standard method for error propagation.

## Supporting Information

Data S1 **Continuous dynamic model generation and perturbation.**
(DOC)

Figure S1 **Expression profiles of miR-200b cluster members upon SNAI1-induction in the MCF7-SNAI1 EMT cell model.** MiR-200b (A), miR-200a (B), miR-429 (C) expression levels were determined by qRT-PCR and normalized to U44 expression and expression levels in non-induced MCF7-SNAI1 cells.
(TIF)

Figure S2 **Ectopic miR-203 expression levels in stably transfected HTB129 cells.** Mir-203 expression levels were determined by qRT-PCR and normalized to U44 expression and expression levels in HTB129-ctrl cells.
(TIF)

Figure S3 **Proliferation curves of and percentage of early apoptotic events in HTB129-ctrl and HTB129-miR203 cells.** A) Representative proliferation curves of HTB129-ctrl and HTB129-miR203 cells. Cell proliferation was assayed over 96 h and quantified using the MTT assay. B) Percentage of early apoptotic HTB129-ctrl and HTB129-miR203 cells, as determined by flow cytometry using AnnexinV/Propidium iodide staining (BD Pharmingen).
(TIF)

Table S1 **Expression data of miRNAs differentially expressed at an established EMT state.** Microarrays were performed at 72 h and 96 h of SNAI1 induction in our EMT cell model. Averaged expression values for each time point were calculated taking into account only replicates which have moduli of log-ratios≥0.5 and t-test p-values≤0.01.
(XLS)

Table S2 **Large-scale analysis of miRNA expressions combining our SNAI1-induced EMT study with epithelial/mesenchymal signatures.** The large-scale analysis combines our microarray analysis results and signatures from four published miRNA microarray analyses of the NCI60 cancer cell line panel. For each analysis, a table provides a list of miRNAs with corresponding expression levels (UP or DOWN), selected with a p-value threshold of 0.01 (from a t-test using the Park et al. classification). This file includes a Venn diagram of the different analyses.
(XLS)

Table S3 **Correlation analysis of miRNA expression levels.** Expression matrices of four published NCI60 studies were processed with the M@IA environment [38], by merging replicates with the average method on the miRNA_id provided in each study (miRBase). Correlation matrices were calculated with the R environment (http://www.r-project.org), with the Pearson method. For each miRNA, the average correlation was calculated, considering correlation with all members of the miR-200 family and allowed to establish a rank correlation (the higher the average correlation, the lower the rank).
(XLS)

Table S4 **Primers used for detection of miRNAs and mRNAs** (primer sequences (5′ – 3′)) (Genecust).
(TIF)
