# Supplementary material for: A Novel Network Integrating a miRNA-203/SNAI1 Feedback Loop which Regulates Epithelial to Mesenchymal Transition
Source: PLoS One. 2012 Apr 13;7(4):e35440. doi: 10.1371/journal.pone.0035440 (PMC3325969; doi:10.1371/journal.pone.0035440)
Supplement: Data S1 — Continuous dynamic model generation and perturbation. (DOC) [file pone.0035440.s001.doc]

**Supplementary Data:**

The stable states of the network were calculated using the SQUAD program [1]. After computing the stable states in a discrete model with an asynchronous updating scheme [2], SQUAD used them in a continuous model to show that the network converges into two unique stable states (supplementary Table a). The probability of transition between the stable states was calculated in the discrete model after modeling the stochastic behavior of the system (GenYsis, [2]) (supplementary Table b), and showed that the probability of the system to spontaneously switch between the stable states after introducing noise according to this model is very low. In other words, the robustness of the system under stochasticity is very high, a common feature in biological networks.

|  | **stable state “E”** | **stable state “M”** |
| --- | --- | --- |
| **CDH1** | 1 | 0 |
| **SNAI1** | 0 | 1 |
| **ZEB1** | 0 | 1 |
| **ZEB2** | 0 | 1 |
| **miR-200** | 1 | 0 |
| **miR-203** | 1 | 0 |

Supplementary Table a. Stable states of the EMT core network. Two unique stable states are present in the system, coined stable state “E” and stable state “M”. The values should be considered normalized in the sense that the range is from 0 to 1 for all genes, and represent the level of activity for this specific gene.

|  | **stable state “E”** | **stable state “M”** |
| --- | --- | --- |
| **stable state “E”** | 0.9935 | 0.0065 |
| **stable state “M”** | 0 | 1 |

Supplementary Table b. Probability of transition matrix. Shown is the probability of transition matrix between stable states after introducing noise in the system according to the stochasticity model. The network exhibits high robustness, with a probability of remaining in the same stable state of 0.9935 and 1 for the state “E” and “M”, respectively.

In order to evaluate the importance of the miR203/SNAI1 double negative feedback loop, we performed a dynamical simulation of a network lacking the interaction “miR-203 on SNAI1”. This edge-altered network converges into one unique stable state (“E_ea_”) (supplementary Table c).

|  | **stable state “E_ea_”** |
| --- | --- |
| **CDH1** | 1 |
| **SNAI1** | 0 |
| **ZEB1** | 0 |
| **ZEB2** | 0 |
| **miR-200** | 1 |
| **miR-203** | 1 |

Supplementary Table c. Stable state of edge-altered network. The system adopts one stable state, coined stable state “E_ea_”. The values should be considered normalized in the sense that the range is from 0 to 1 for all genes, and represent the level of activity for this specific gene.

**References**

1. Di Cara A, Garg A, De Micheli G, Xenarios I, Mendoza L (2007) Dynamic simulation of regulatory networks using SQUAD. BMC Bioinformatics 8: 462.

2. Garg A, Mohanram K, Di Cara A, De Micheli G, Xenarios I (2009) Modeling stochasticity and robustness in gene regulatory networks. Bioinformatics 25(12): i101-109.
