# Supplementary figures and images for: A Novel Network Integrating a miRNA-203/SNAI1 Feedback Loop which Regulates Epithelial to Mesenchymal Transition
Source: PLoS One. 2012 Apr 13;7(4):e35440. doi: 10.1371/journal.pone.0035440 (PMC3325969; doi:10.1371/journal.pone.0035440)

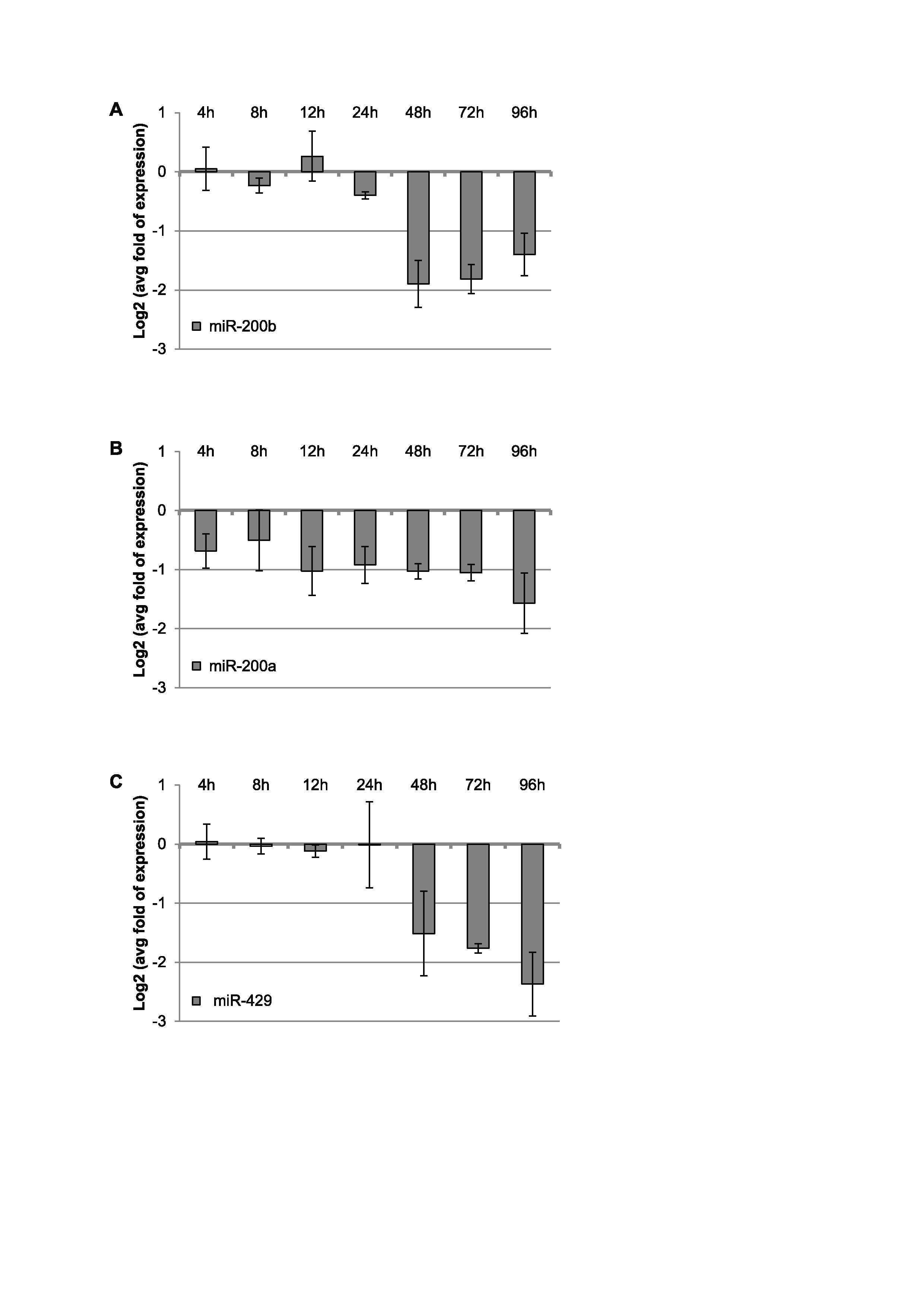

Supplement: Figure S1 — Expression profiles of miR-200b cluster members upon SNAI1-induction in the MCF7-SNAI1 EMT cell model. MiR-200b (A), miR-200a (B), miR-429 (C) expression levels were determined by qRT-PCR and normalized to U44 expression and expression levels in non-induced MCF7-SNAI1 cells. (TIF) [file pone.0035440.s002.tif]

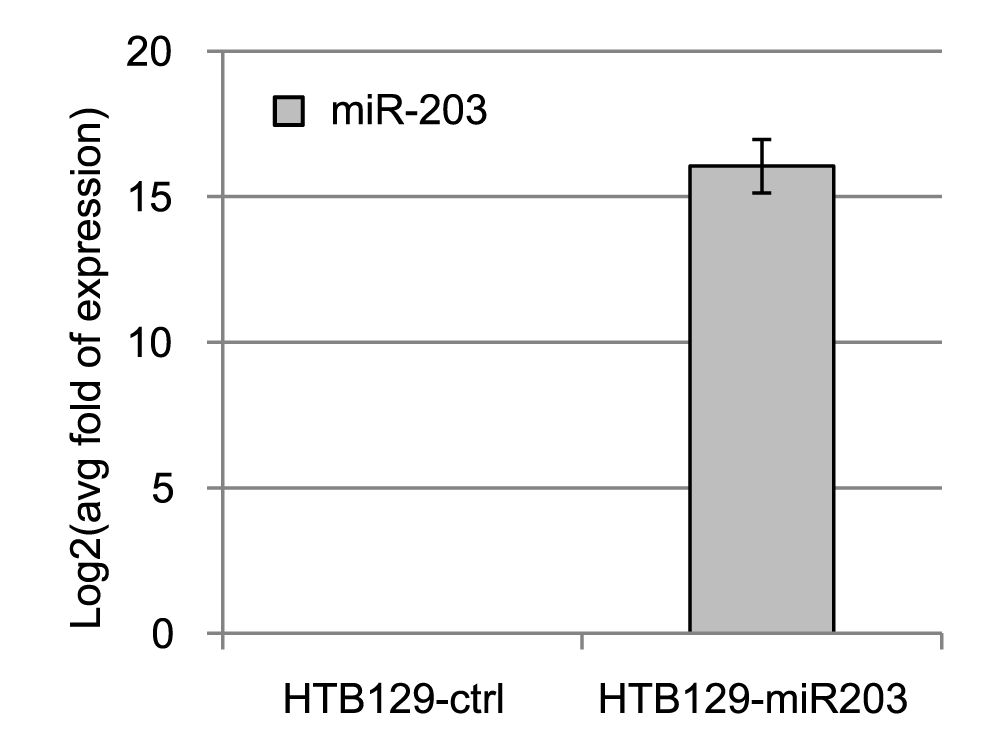

Supplement: Figure S2 — Ectopic miR-203 expression levels in stably transfected HTB129 cells. Mir-203 expression levels were determined by qRT-PCR and normalized to U44 expression and expression levels in HTB129-ctrl cells. (TIF) [file pone.0035440.s003.tif]

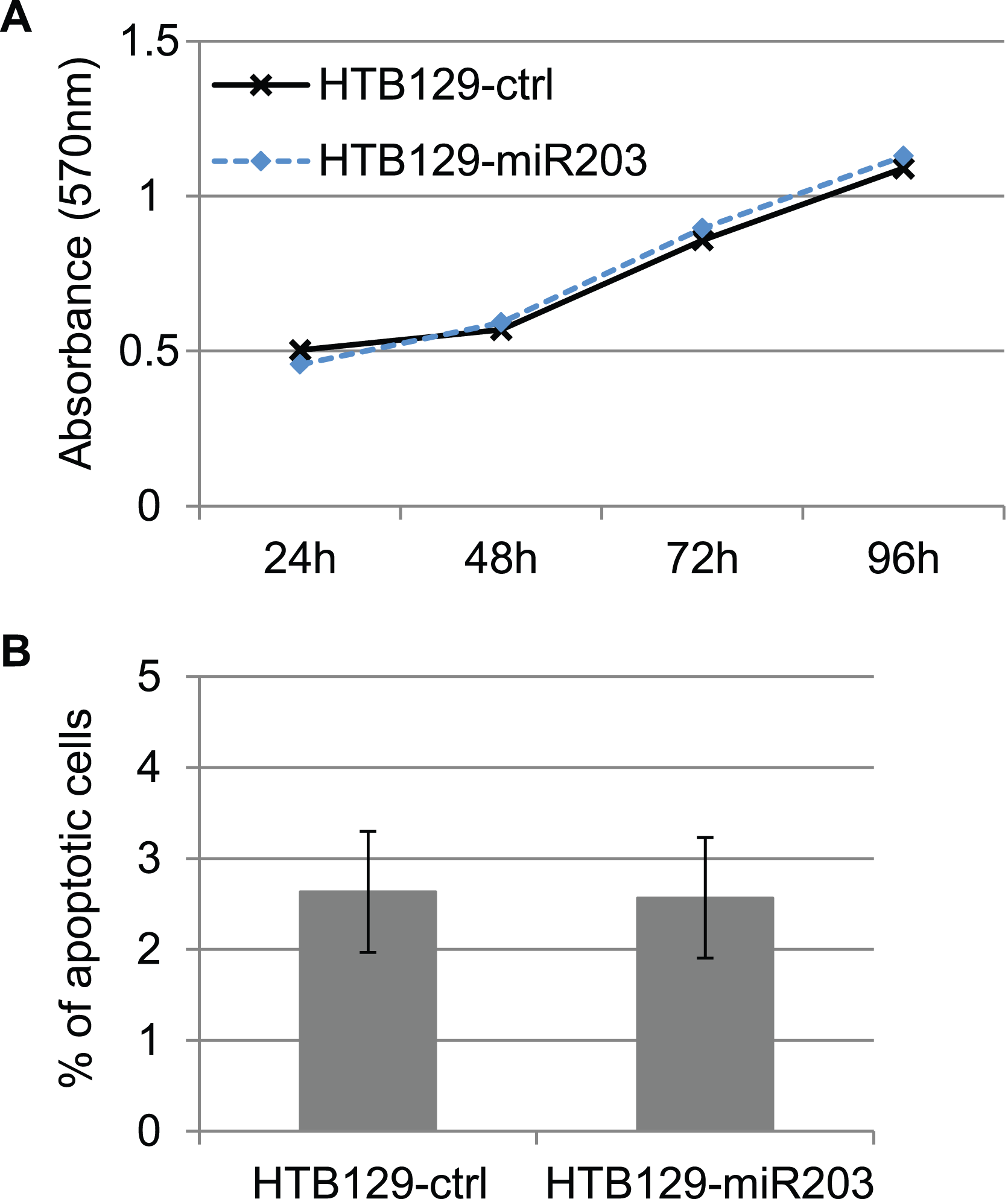

Supplement: Figure S3 — Proliferation curves of and percentage of early apoptotic events in HTB129-ctrl and HTB129-miR203 cells. A) Representative proliferation curves of HTB129-ctrl and HTB129-miR203 cells. Cell proliferation was assayed over 96 h and quantified using the MTT assay. B) Percentage of early apoptotic HTB129-ctrl and HTB129-miR203 cells, as determined by flow cytometry using AnnexinV/Propidium iodide staining (BD Pharmingen). (TIF) [file pone.0035440.s004.tif]

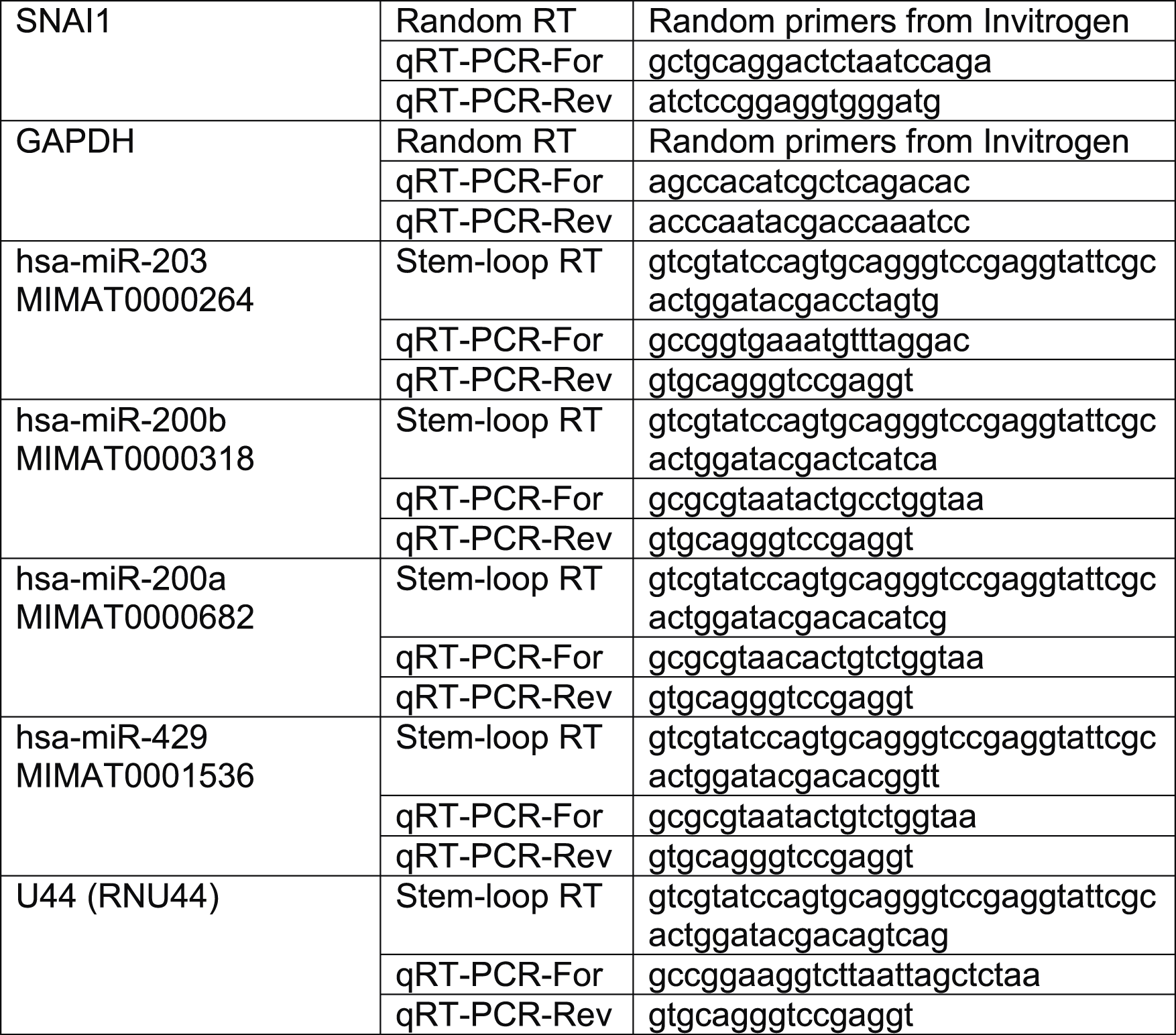

Supplement: Table S4 — Primers used for detection of miRNAs and mRNAs (primer sequences (5′ – 3′)) (Genecust). (TIF) [file pone.0035440.s008.tif]
